# Manifold regularization for sparse unmixing of hyperspectral images

**DOI:** 10.1186/s40064-016-3671-6

**Published:** 2016-11-24

**Authors:** Junmin Liu, Chunxia Zhang, Jiangshe Zhang, Huirong Li, Yuelin Gao

**Affiliations:** 1 School of Mathematics and Statistics, Xi’an Jiaotong University, Xianning West Road, Xi’an, 710049 China; 2 School of Information and Computing Science, Beifang University of Nationalities, Wenchang North Road, Yinchuan, 750021 China

**Keywords:** Hyperspectral images, Spectral unmixing, Manifold regularization

## Abstract

**Background:**

Recently, *sparse unmixing* has been successfully applied to spectral mixture analysis of remotely sensed hyperspectral images. Based on the assumption that the observed image signatures can be expressed in the form of linear combinations of a number of pure spectral signatures known in advance, unmixing of each mixed pixel in the scene is to find an optimal subset of signatures in a very large spectral library, which is cast into the framework of sparse regression. However, traditional sparse regression models, such as *collaborative sparse regression*, ignore the intrinsic geometric structure in the hyperspectral data.

**Results:**

In this paper, we propose a novel model, called *manifold regularized collaborative sparse regression*, by introducing a manifold regularization to the collaborative sparse regression model. The manifold regularization utilizes a graph Laplacian to incorporate the locally geometrical structure of the hyperspectral data. An algorithm based on *alternating direction method of multipliers* has been developed for the manifold regularized collaborative sparse regression model.

**Conclusions:**

Experimental results on both the simulated and real hyperspectral data sets have demonstrated the effectiveness of our proposed model.

## Background

In recent years, remotely sensed hyperspectral images have been widely used for various applications ranging from civilian to military purposes (Somers and Delalieux 2009; Settle and Drake 1993; Chang and Heinz 2000; Shimabukuro and Carvalho 1997), since they can provide abundant wavelength information of the land covers with spectral resolution at the micron level (Shaw and Burke 2003). For example, the National Aeronautics and Space Administration (NASA) Hyperion sensor on Earth Observing-1 (EO-1) satellite can provide hyperspectral images with 220 bands and a spectral resolution of the order of 10 nm. Despite high spectral resolution, the relatively low spatial resolution of hyperspectral images leads to *mixed pixel* problem, i.e., a single pixel usually contains several distinct materials. The existence of mixed pixels seriously restricts the application of hyperspectral images. To cope with mixed pixels, *spectral unmixing* (Keshava 2003; Bioucas-Dias and Plaza 2012; Meer 2012), which aims at decomposing the observed mixed pixel spectrum into a collection of pure substance spectra, namely *endmembers*, and their corresponding fractional *abundances*, has been proposed and widely applied in hyperspectral remote sensing data analysis (Bioucas-Dias and Plaza 2013).

For spectral unmixing, two kinds of models—the *linear mixture model* (LMM) (Singer et al. 1979) and the *non-linear mixture model* (NMM) (Hapke 1981)—have been proposed to characterize the mixed pixels. Although the LMM is not as accurate as the NMM to capture the mixing behavior of mixed pixels, it is more popular than the NMM for solving the spectral unmixing problem because of its simplicity and efficiency in most cases (Fan et al. 2009). In addition, the rapidly developed methods in classical signal processing field also provide effective tools to the solution of the LMM (Ma et al. 2014). Nevertheless, modeling the mixed pixels is a very complex and difficult task. In practice, we have to make a compromise between model accuracy and tractability. Therefore, we here focus on the LMM in this study.

The standard LMM used for spectral unmixing assumes that each pixel spectrum is a linear combination of the endmembers present in the scene weighted by the corresponding abundances. From the convex geometry point of view, the LMM forces the mixed pixels to belong to a simplex (or a convex hull), and the vertices of simplex correspond to the endmembers. Based on the geometrical interpretation, many spectral unmixing algorithms have been proposed for endmember extraction such as the N-FINDR (Winter 1999), *pixel purity index* (PPI) (Boardman et al. 1995), *vertex component analysis* (VCA) (Nascimento and Bioucas-Dias 2005), *simplex growing algorithm* (SGA) (Chang 2006) and their variants (Chan et al. 2011; Liu and Zhang 2012; Chang et al. 2010), and for abundance estimation such as the *fully constrained least squares* (FCLS) (Heinz and Chang 2001), *distance geometry-based abundance estimation* (DGAE) (Pu et al. 2014), and so on. However, these geometrical-based algorithms are likely to fail when the pixels are highly mixed. As an alternative, the statistical algorithms have been developed by formulating the spectral unmixing as a statistical inference problem. Such algorithms include the *dependent component analysis* (DECA) (Nascimento and Bioucas-Dias 2012), *beta compositional model* (BCM) (Zare et al. 2013) and *normal compositional model* (NCM) (Stein 2003) methods. Although the statistical algorithms have a natural framework for incorporating various priors and *endmember variability* (Somers et al. 2011; Zare and Ho 2014), it is difficult to derive the close-form expressions of the inference parameters and thus they suffer from high computational complexity.

Most of the spectral unmixing algorithms based on the standard LMM can not automatically determine the number of endmembers present in the scene. In addition, some endmembers produced by these algorithms are not necessarily present in the image, producing the so-called *virtual* endmembers (Chen 2011). The virtual endmembers can compensate the approximation of the LMM but will result in unidentifiability of the materials. To tackle these problem, the standard LMM has been extended into a semisupervised version (Liu and Zhang 2014; Iordache et al. 2012; Zhong and Zhang 2014; Feng et al. 2014; Iordache et al. 2011), i.e., by assuming that the endmembers are known in advance. Typically, Iordache et al. (2011) have proposed the *sparse regression* (SR) model by assuming that the endmembers present in the scene belong to a subset of samples available a *priori* in a library. The unmixing based on SR is called *sparse unmixing*. Experimental results have illustrated the potential of sparse unmixing in abundance estimation. The success of sparse unmixing relies crucially on the availability of suitable hyperspectral libraries because the libraries are hardly acquired under the same conditions of the remotely sensed images. Fortunately, this problem can be overcome by a delicate calibration procedure to adapt the library to the image or learning of the libraries directly from the data set without other *priori* information (Charles et al. 2011). The SR problem can be efficiently solved via the *sparse unmixing algorithm via variable splitting and augmented Lagrangian* (SUnSAL) (Iordache et al. 2011; Bioucas-Dias and Figueiredo 2010) by exploiting the sparse prior induced by the $\ell _1$ norm. However, a high correlation of the spectral signatures limits the unmixing accuracy. To mitigate this limitation, Iordache et al. (2014a) have developed a *collaborative* SR (CSR) model by considering the structured sparsity, which exploits the fact that only a few spectral signatures in the library are active, in other words, only a few lines of abundances collected in a matrix are nonzero. Some modifications of the CSR can be found in Iordache et al. (2014b), Tang et al. (2015). However, the improvements in Iordache et al. (2014b), Tang et al. (2015) are limited since only the spectral information is considered to estimated the abundances.

Generally, the size of spectral library is often large, while the number of endmembers present in the scene is very small. Therefore, the fractional abundances are more likely to reside on a low-dimensional submanifold of the high-dimensional ambient Euclidean space. However, existing sparse unmixing methods only consider the Euclidean structure of the data space while ignoring the intrinsic manifold structure of the hyperspectral data. Many previous studies (Lu et al. 2013; Zheng et al. 2011; Guan et al. 2011; Seung and Lee 2000; He and Niyogi 2004; Belkin et al. 2006) have shown that exploiting the local geometrical structure (i.e., the intrinsic manifold structure) is very important to the model learning and data representation. In this paper, we incorporate the *manifold regularization* (Belkin et al. 2006) to the CSR and develop a novel model, called *manifold regularized collaborative sparse regression* (MCSR) model. The manifold regularization is characterized by a *Laplacian graph* which captures the local geometrical structure of the data manifold such that nearby mixed pixels in the intrinsic geometry of the data space are likely to have similar fractional abundances. By adding an additional manifold structure learning term to CSR, our proposed MCSR model is expected to have higher unmixing accuracy than CSR. To solve the MCSR, an optimization algorithm based on *alternating direction method of multipliers* (ADMM) is developed. It should be noted that similar works of using the manifold regularization have also been introduced in Lu et al. (2013), Tong et al. (2014), but they are different with ours in the following two main aspects. First, the works in Lu et al. (2013), Tong et al. (2014) are based on the standard LMM while our model is an extension of the SR model. Second, multiplicative iterative algorithm is used to optimize the nonnegative matrix factorization model in Lu et al. (2013), Tong et al. (2014), whereas the ADMM is used to cope with the proposed MCSR model.

## Related work

In this section, we first describe the sparse unmixing problem and then briefly review the CSR model.

### Sparse unmixing

The standard LMM signors the intimate mixture and holds at a macroscopic level (Bioucas-Dias and Plaza 2012). For a pixel spectrum ${y}\in {\mathscr {R}}^l$ with *l* spectral bands, LMM assume that it is a linear combination of the endmembers present in the scene weighted by the corresponding abundances, i.e.,

1 $$\begin{aligned} {y}=\sum _{i=1}^p{m}_i\alpha _i+{e}\end{aligned}$$

2 $$\begin{aligned}={M}\alpha +{e}, \end{aligned}$$

where ${\alpha }=(\alpha _1,\ldots ,\alpha _p)^T\in {\mathscr {R}}^p$ is the abundance vector, ${M}\in {\mathscr {R}}^{l\times p}$ is the endmember matrix with each column ${m}_i\in {\mathscr {R}}^l$ being an endmember present in the scene, ${e}\in {\mathscr {R}}^l$ denotes the noise or error term, and *p* is the number of endmembers present in the scene. To be physically meaningful, the abundance vector is usually *subject to* (*s.t.*) the *sum-to-one* and *nonnegativity* constraints

3 $$\begin{aligned}(\text {sum-to-one}):\quad \sum _{i=1}^p\alpha _i=1, \end{aligned}$$

4 $$\begin{aligned}(\text {nonnegativity}):\quad \alpha _i\ge 0,\quad i=1,2,\ldots ,p. \end{aligned}$$

Over the past decades, lots of spectral unmixing algorithms (Keshava 2003; Bioucas-Dias and Plaza 2012, 2013) have been developed based on the above LMM. However, the abundance estimation by these algorithms usually relies on the availability of pure spectral signatures in the input data or on their capacities of extracting endmembers. In addition, some algorithms perform unmixing by assuming all of the endmembers in ${M}$ are present in the scene and by exploiting the sparsity prior of abundance ${\alpha }$. If the abundance ${\alpha }$ is not sparse or sufficiently sparse, the results obtained by these algorithms will not be as accurate as we expect. To cope with these problems, *sparse unmixing* (Iordache et al. 2011) has been introduced based on the assumption that the endmember set $\{{m}_1,{m}_2,\ldots ,{m}_p\}$ present in the scene is contained in a spectral library denoted by $\{{a}_1,{a}_2,\ldots ,{a}_m\}$ known in advance, i.e., $\{{m}_1,{m}_2,\ldots ,{m}_p\}\subset \{{a}_1,{a}_2,\ldots ,{a}_m\}$. With the ever-growing availability of spectral libraries, the number of endmembers present in the scene is much less than the total number of endmembers in spectral library; thus, we have $p\ll m$. By this way, unmixing is to find the optimal subset of signatures for the mixed pixels in the spectral library. The sparse unmixing model can be written as

5 $$\begin{aligned} {y}={A}{x}+{e}, \end{aligned}$$

where ${A}=[{a}_1,{a}_2,\ldots ,{a}_m]\in {\mathscr {R}}^{l\times m}$ is the spectral library, and ${x}\in {\mathscr {R}}^{m}$ is the abundance vector corresponding the spectral library ${A}$. Clearly, ${x}$ is sparse. By exploiting the sparse prior of abundance ${x}$ through the well-known $\ell _1$ norm, we can estimate abundance vector ${x}$ by the following *sparse regression* (SR) problem:

6 $$\begin{aligned} \min _{{x}}\frac{1}{2}\Vert {y}-{A}{x}\Vert _2^2+\lambda _{\text {SR}}\Vert {x}\Vert _1\quad s.t.\;{x}\ge {0}, \end{aligned}$$

where $\Vert {x}\Vert _1=\sum _{i=1}^m|x_i|$ and $\lambda _{\text {SR}}>0$ is a sparse regularization parameter. It is worth to note that the sum-to-one constraint is no longer necessary since the nonnegativity constraint can automatically lead to the sum-to-one constraint as stated in Bioucas-Dias and Plaza (2012), Iordache et al. (2011). However, the high mutual coherence of the spectral library ${A}$ limits the unmixing accuracy.

### Collaborative sparse regression

In fact, sparse unmixing, as a semi-supervised model, is a typical underdetermined linear system. To solve it, sparsity prior for the fractional abundance of each individual pixel is imposed in (6). Although the high level of sparsity of fractional abundances can enhance the recovery ability of the $\ell _1$-*minimization* problem (6), the highly correlated samples in library still restrict the capability of model (6) to obtain a desirable solution. To tackle this problem, the CSR model has been recently proposed in Iordache et al. (2014a). Unlike the $\ell _1$-*minimization* problem (6), the CSR model simultaneously (or collaboratively) imposes a sparsity to all pixels in the data set by exploiting the fact that pixels in a scene should share the same set of active endmembers, and thus only a few rows of the abundance matrix are nonzero.

Let ${Y}=[{y}_1,{y}_2,\ldots ,{y}_n]\in {\mathscr {R}}^{l\times n}$ be the *n* observed pixels arranged in a matrix, we can rewrite the sparse unmixing model (5) in the matrix form as

7 $$\begin{aligned} {Y}={A}{X}+{E}, \end{aligned}$$

where ${E}=[{e}_1,{e}_2,\ldots ,{e}_n]\in {\mathscr {R}}^{l\times n}$ is the noise matrix and ${X}=[{x}_1,{x}_2,\ldots ,{x}_n]\in {\mathscr {R}}^{m\times n}$ is the fractional abundance matrix. With the collaborative sparsity induced by the row-sparsity regularizer $\ell _{2,1}$ norm, the CSR problem can be formulated as

8 $$\begin{aligned} \min _{{X}}\frac{1}{2}\Vert {Y}-{A}{X}\Vert _F^2+\lambda _{\text {CSR}}\Vert {X}\Vert _{2,1}\quad s.t.\,{X}\ge {0}, \end{aligned}$$

where $\Vert {X}\Vert _{2,1}=\sum _{i=1}^m\Vert {x}^i\Vert _2$, ${x}^i$ represents the *i*th row of abundance fraction matrix ${X}$ and $\lambda _{\text {CSR}}$ is a regularization parameter. An algorithm called *collaborative sparse unmixing via variable splitting and augmented Lagrangian* (CLSUnSAL) is provided in Iordache et al. (2014a) and a number of experiments have shown that the imposed collaborative sparsity prior can significantly reduce the probability of recovery failure.

## Methods

In this section, we introduce an enhanced CSR model, called *manifold regularized* CSR (MCSR) model, by incorporating a manifold regularization to CSR, and then an alternating direction method is developed to solve the resulting optimization problem.

### MCSR

As for CSR problem (8), the data fitting term $\Vert {Y}-{AX}\Vert _F^2$ is useful for learning the Euclidean structures in the hyperspectral data space. As we have previously mentioned, the size of spectral library is usually very large, while the number of endmembers present in the scene is very small. From a geometric viewpoint, the fractional abundances are more likely to reside on a low-dimensional submanifold of the high-dimensional ambient Euclidean space. Recent studies (Lu et al. 2013; Zheng et al. 2011; Guan et al. 2011; Seung and Lee 2000; He and Niyogi 2004; Belkin et al. 2006) have shown that intrinsic geometric structures on manifolds are very important to the data representation and many manifold learning methods (Tenenbaum et al. 2000; Roweis and Saul 2000; Donoho and Grimes 2003; Belkin and Niyogi 2003; Lin and Zha 2008) have been proposed to recover the geometry of a data set. In the literature of spectral unmixing, many existing methods (Lu et al. 2013) only explore the Euclidean structure while fail to discover the intrinsic geometry structure of the data manifold. Therefore, we want to explore the ability of the intrinsic geometry structure of the hyperspectral data in improving the unmixing accuracy.

To preserve the intrinsic geometry structure of the data, a natural assumption, referred to the *manifold assumption* (He and Niyogi 2004), is that nearby data points are also nearby points in their low-dimensional representations. However, modeling the global geometric structures of the data is a very big challenge due to the insufficient number of samples and the high dimensionality of the ambient space. In practice, a nearest neighbor graph on the data points is often used to characterize the underlying local geometric structures.

Given *n* data points $\{{y}_1,{y}_2,\ldots ,{y}_n\}\in {\mathscr {R}}^l$ sampled from the underlying submanifold, we construct a nearest neighbor graph ${\mathscr {G}}$ with its *i*th node corresponding to the data point ${y}_i, i=1,2,\ldots ,n$. For each node ${y}_i$, one can put an edge between it and its *k* nearest neighbors. Let ${\mathscr {N}}_k({y}_i)=\{{y}_i^1,\ldots ,{y}_i^k\}$ be the set of its *k* nearest neighbors. And we define the weight matrix ${W}$ on the graph ${\mathscr {G}}$ as

$$\begin{aligned} w_{ij}=\left\{ \begin{array}{ll} \frac{{y}_i^T{y}_j}{\Vert {y}_i\Vert _2^2\cdot \Vert {y}_j\Vert _2^2},&{}\quad {\text {if}}\; {y}_{i}\in {\mathscr {N}}_k({y}_j)\;{\text {or}}\;{y}_j\in {\mathscr {N}}_k({y}_i),\\ 0,&{}\quad \text {otherwise}. \end{array} \right. \end{aligned}$$

The weight $w_{ij}$ is used to measure the similarity between data points ${y}_i$ and ${y}_j$, other similarity measures can also be used to evaluate the similarity (Cai et al. 2011). For the manifold assumption, i.e., if two points ${y}_i$ and ${y}_j$ are close to each other, then their low-dimensional representations ${x}_i$ and ${x}_j$ are close as well, a natural choice is to minimize the following manifold regularization ${\mathscr {T}}$, defined by

9 $$\begin{aligned} {\mathscr {T}}&=\frac{1}{2}\sum _{i=1}^n\sum _{j=1}^nw_{ij}\Vert {x}_i-{x}_j\Vert ^2_2\nonumber \\&=\text {Tr}({X}{L}{X}^T) \end{aligned}$$

where ${L}={D}-{W}$ and ${D}$ is a diagonal matrix with the *i*th diagonal element $d_{ii}=\sum _{j}w_{ij}$. The matrix ${L}$ is usually called *graph Laplacian* (Cai et al. 2011; He et al. 2011). It is apparent that minimizing the manifold regularization ${\mathscr {T}}$ imposes smoothness of the representation coefficients, or equally the prior assumption that if neighboring points ${y}_i$ and ${y}_j$ are similar (with a relatively bigger weight $w_{ij}$), their low-dimensional representations ${x}_i$ and ${x}_j$ should be very close. Therefore, minimizing (9) is an attempt to ensure the manifold assumption.

By incorporating the above manifold regularization ${\mathscr {T}}$ into the CSR, we have the MCSR problem as

10 $$\begin{aligned} \min _{{X}}\,&\frac{1}{2}\Vert {Y}-{A}{X}\Vert _F^2+\lambda _{\text {CSR}}\Vert {X}\Vert _{2,1}+\frac{1}{2}\lambda _{\text {MR}}{\mathscr {T}}\\&s.t.\;{X}\ge {0},\nonumber \end{aligned}$$

where $\lambda _{\text {MR}}>0$ is a manifold regularization parameter. The problem (10) has the following equivalent form

11 $$\begin{aligned} \min _{{X}}\,\frac{1}{2}\Vert {Y}-{A}{X}\Vert _F^2+\lambda _{\text {CSR}}\Vert {X}\Vert _{2,1}+\frac{1}{2}\lambda _{\text {MR}}{\mathscr {T}}+\iota _{{\mathscr {R}}_+}({X}) \end{aligned}$$

where $\iota _{{\mathscr {R}}_+}$ is an indicator function, defined by

12 $$\begin{aligned} \iota _{{\mathscr {R}}_+}(x)=\left\{ \begin{array}{ll} 0,&{}\quad {\text {if}}\;x\ge 0,\\ +\infty ,&{}\quad {\text {otherwise}}. \end{array} \right. \end{aligned}$$

### Application of ADMM to MCSR

In this subsection, we propose to apply the *alternating direction method of multipliers* (ADMM) (Afonso et al. 2011; Yang and Zhang 2011) method to the MCSR problem (10). The ADMM method has recently attracted more attention because it can decouple the variables, and it is usually used to solve the problems of a convex, non-smooth objective function with structured linear constraints.

Consider the following structured optimization problem with linear constraints:

13 $$\begin{aligned} \min _{{x},{v}}\,f({x})+g({v})\quad s.t.\,\,{G}{x}={v}, \end{aligned}$$

where both $f({x})$ and $g({v})$ are convex functions, ${G}$ is a known matrix with full column rank. For this problem, the augmented Lagrangian function is given by

$$\begin{aligned} {\mathscr {L}}({x},{v},{\alpha })&=f({x})+g({v})+{\alpha }^T({G}{x}-{v})+\frac{\mu }{2}\Vert {Gx}-{v}\Vert ^2_2\\&=f({x})+g({v})+\frac{\mu }{2}\Vert {G}{x}-{v}-{d}\Vert _2^2+{\text {constant}} \end{aligned}$$

where ${\alpha }$ is the Lagrange multipliers, $\mu >0$ is a penalty parameter, and ${d}=-{\alpha }/\mu$. ADMM alternately minimizes ${\mathscr {L}}({x},{v},{\alpha })$ with respect to ${x}$ and ${v}$ in a Gauss–Seidel manner. The general procedures of ADMM are summarized in Algorithm 1.
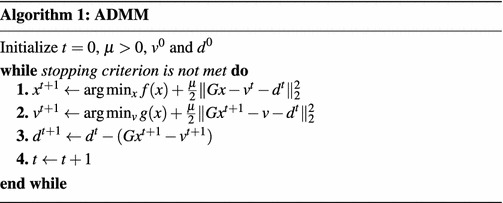


The convergence of ADMM is guaranteed by the following theorem given in Eckstein and Bertsekas (1992) (see Theorem 8).

#### **Theorem 1**

*Consider problem* (13) *with* ${G}$ *having full columns rank and* *f*, *g* *being closed, proper, convex functions. Then, for arbitrary* $\mu >0$ *and* ${x}^0, {v}^0, {d}^0$, *if problem* (13) *has a solution, the* *sequences* $\{{x}^t,{v}^t,{d}^t\}$ *generated by Algorithm 1 converges to it; otherwise, at least one of the sequences* $\{{d}^t\}$ *and* $\{({x}^t,{v}^t)\}$ *diverges.*

According to the above framework of ADMM, and let

14 $$\begin{aligned}{G}{X}=\left[ \begin{array}{c} {I}\\ {I} \end{array}\right] {X}= \left[ \begin{array}{c} {V}_1\\ {V}_2 \end{array}\right] ={V}, \end{aligned}$$

15 $$\begin{aligned}f({X})=\frac{1}{2}\Vert {Y}-{A}{X}\Vert _F^2, \end{aligned}$$

where ${I}$ is an identity matrix, and

16 $$\begin{aligned} g({V})=\lambda _{\text {CSR}}\Vert {V}_1\Vert _{2,1}+\iota _{{\mathscr {R}}_+}({V}_1)+\frac{\lambda _{\text {MR}}}{2}\text {Tr}\left( {V}_2{L}{V}_2^T\right) , \end{aligned}$$

we have the corresponding augmented Lagrangian function as

$$\begin{aligned}&{\mathscr {L}}({X},{V}_1,{V}_2,{D}_1,{D}_2)\nonumber \\&\quad =f({X})+g({V})+\frac{\mu }{2}\Vert {G}{X}-{V}-{D}\Vert _F^2\\&\quad =\frac{1}{2}\Vert {Y}-{A}{X}\Vert _F^2+\lambda _{\text {CSR}}\Vert {V}_1\Vert _{2,1}+\iota _{{\mathscr {R}}_+}({V}_1)+\frac{\lambda _{\text {MR}}}{2}\text {Tr}\left( {V}_2{L}{V}_2^T\right) \\&\qquad +\frac{\mu }{2}\Vert {X}-{V}_1-{D}_1\Vert _F^2+\frac{\mu }{2}\Vert {X}-{V}_2-{D}_2\Vert _F^2. \end{aligned}$$

Then, we apply the alternating minimization idea to update the variables ${X}, {V}_1, {V}_2$ and the Lagrange multipliers ${D}_1, {D}_2$. Given the current point ${X}^t, {V}_1^t$, ${V}_2^t$, ${D}_1^t, {D}_2^t$, we get the next step of ${X}$ by minimizing ${\mathscr {L}}$ with respect to ${X}$, i.e.,

$$\begin{aligned} {X}^{t+1}&\in \arg \min _{{X}}f({X})+\frac{\mu }{2}\left\| {G}{X}-{V}_1^t-{D}_2^t\right\| _2^2\\&\in \arg \min _{{X}}\frac{1}{2}\Vert {Y}-{A}{X}\Vert _F^2+\frac{\mu }{2}\left\| {X}-{V}_1^t-{D}_1^t\right\| _F^2+\frac{\mu }{2}\left\| {X}-{V}_2^t-{D}_2^t\right\| _F^2, \end{aligned}$$

which yields the following updating rule

$$\begin{aligned} {X}^{t+1}=({A}^T{A}+2\mu {I})^{-1}({A}^T{Y}+\mu \zeta ^t), \end{aligned}$$

where $\zeta ^t={V}_1^t+{V}_2^t+{D}_1^t+{D}_2^t$. To update ${V}_1$ and ${V}_2$, we have the augmented Lagrangian subproblems

$$\begin{aligned} {V}_1^{t+1}&\in \arg\min _{{V}_1}\lambda _{\text {CSR}}\Vert {V}_1\Vert _{2,1}+\frac{\mu }{2}\left\| {X}^{t+1}-{V}_1-{D}_1^{t}\right\| _F^2+\iota _{{\mathscr {R}}_+}({V}_1)\\ {V}_2^{t+1}&\in \arg\min _{{V}_2}\frac{\lambda _{\text {MR}}}{2}\text {Tr}\left( {V}_2{L}{V}_2^T\right) +\frac{\mu }{2}\left\| {X}^{t+1}-{V}_2-{D}_2^{t}\right\| _F^2. \end{aligned}$$

Before solving ${V}_1$, we first define the well-known *vector-soft thresholding* (VST) operator ${\mathscr {V}}_{\tau }$ of a matrix ${Q}=[{q}_1^T,{q}_2^T,\ldots ,{q}_m^T]^T\in {\mathscr {R}}^{m\times n}$ as

17 $$\begin{aligned} {\mathscr {V}}_{\tau }({Q})={Q}^*, \end{aligned}$$

where the *i*th row of ${Q}^*$ is ${q}_i^*$, defined by

18 $$\begin{aligned} {q}^*_i=\left\{ \begin{array}{ll} \frac{\Vert {q}_i\Vert -\tau }{\Vert {q}_i\Vert }{q}_i,&{}\quad {\text {if}}\;\tau <\Vert {q}_i\Vert ,\\ {0},&{}\quad {\text {otherwise}}. \end{array} \right. \end{aligned}$$

By the VST operator, it is easy to get the updating rule of ${V}_1$ as

19 $$\begin{aligned} {V}_1^{t+1}=\max \left( {0},{\mathscr {V}}_{\lambda _{\text {CSR}}/\mu }\left( {X}^{t+1}-{D}_1^t\right) \right) . \end{aligned}$$

As for ${V}_2$, we have

20 $$\begin{aligned} {V}^{t+1}_2=\left( {X}^{t+1}-{D}_2^t\right) \left( \frac{\lambda _{\text {MR}}}{\mu }{L}+{I}\right) ^{-1}. \end{aligned}$$

The Lagrangian multipliers ${D}_1, {D}_2$ can be updated as

$$\begin{aligned} {D}_1^{t+1}&={D}_1^{t}-\left( {X}^{t+1}-{V}_1^{t+1}\right) ,\\ {D}_2^{t+1}&={D}_2^{t}-\left( {X}^{t+1}-{V}_2^{t+1}\right) . \end{aligned}$$

Finally, the proposed *manifold regularized collaborative sparse unmixing* via ADMM (MCSUnADMM) algorithm for MCSR is summarized in Algorithm 2.
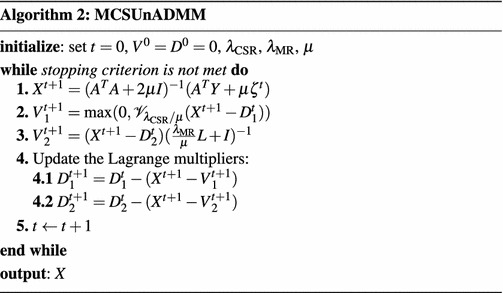


The convergence of Algorithm 2 is guaranteed by Theorem 1, since it can be expressed as an instance of problem (13). ${G}$ is a full column rank matrix, and functions *f*, *g* are closed, proper, convex. These meet the conditions in Theorem 1, and hence the convergence of Algorithm 2 is guaranteed.

## Experiments

In this section, we evaluate the performance of our proposed MCSUnADD algorithm, and compare it with the CLSUnSAL algorithm (Iordache et al. 2014a) for the CSR problem (8), the SUnSAL algorithm (Iordache et al. 2011) for the SR problem (6), and the total variation regularized SUnSAL (TVSUnSAL) algorithm (Iordache et al. 2012). Experiments are carried out on two simulated hyperspectral data sets and one real hyperspectral data set, and the accuracy assessment of all the experiments is made by computing the *signal to reconstruction error* (SRE) (Iordache et al. 2011), defined by

21 $$\begin{aligned} \text {SRE}=\frac{\mathbb {E}(\Vert {x}\Vert _2^2)}{\mathbb {E}\left( \Vert {x} -\hat{{x}}\Vert _2^2\right) } \end{aligned}$$

where $\mathbb {E}({x})$ is the expectation of ${x}$ and $\hat{{x}}$ is the corresponding reconstructed abundance vector. The higher the SRE, the better the reconstruction. In our experiments, the regularization parameters $\lambda _{\text {SR}}, \lambda _{\text {CSR}}$ and $\lambda _{\text {MR}}$ are selected from

$$\begin{aligned} \{1e^{-4},5e^{-4},1e^{-3},5e^{-3},0.001,0.005,0.01,0.05,0.1,0.5,1\} \end{aligned}$$

to obtain the best unmixing accuracy. As for stopping criterion, we set as the maximum iteration number and the Frobenius norm of reconstruction errors of the pixels, i.e. $\Vert {Y}-{A}{X}\Vert _F^2$, less than $\tau =1e^{-4}$.

### Synthetic image experiments

Our experiments are first performed on two synthetic images, which were generated from the USGS library ${A}\in {\mathscr {R}}^{224\times 445}$ that contains 445 materials with each having 224 spectral bands that uniformly distribute in the interval 0.4–2.5 μm.

#### *Synthetic Image 1 (SI-1)*

**Fig. 1:**
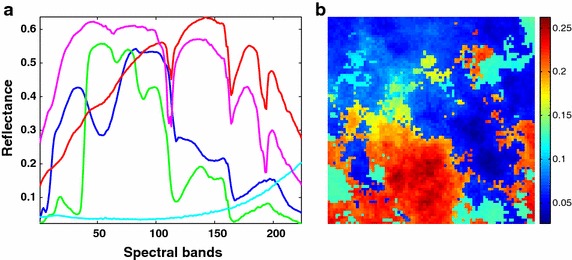
Five randomly selected endmember spectra to generate SI-1 and the corresponding simulated image with band 50. **a** Five endmember spectra and **b** synthetic image with band 50

This synthetic image of size $80 \times 80$ is produced by a hyperspectral imagery synthesis tools, which is developed by the computational intelligence group of the Basque Country University. Five endmembers as shown in Fig. 1a are randomly selected from the spectral library ${A}$ to generate the synthetic image, as shown in Fig. 1b. In this image, the fractional abundances are created by the *Gaussian fields* method with *spheric* type (refer to Boris 1999 for more details) to model the natural scene such as the distribution of forest, and satisfy the LMM, together with the nonnegative and sum-to-one constraints.

#### *Synthetic Image 2 (SI-2)*

**Fig. 2:**
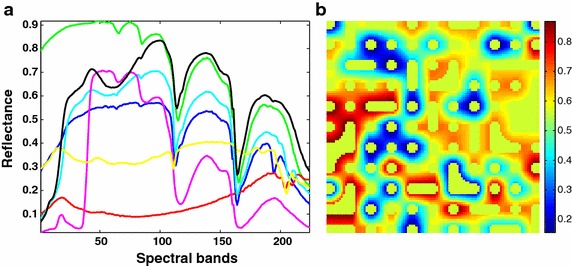
Seven randomly selected endmember spectra to generate SI-2 and the corresponding simulated images with band 50. **a** Seven endmember spectra and **b** synthetic image with band 50

This synthetic image is to simulate a scene with land covers arranged in discrete patches, and was designed in Miao and Qi (2007). In the image, seven endmembers, randomly selected from the spectral library ${A}$ and as shown in Fig. 2a, are used to produce a hyperspectral image with $100\times 100$ pixels. The image is first divided into several $10\,\text {pixels}\times 10\,\text {pixels}$ regions with each initialized with one of the seven endmember spectra, and then a $8\,\text {pixels}\times 8\,\text {pixels}$ spatial low-pass filter is used to generate the mixed pixels. In order to model the scene without pure pixels, all of the pixels whose abundances are larger than 0.8 are replaced with a mixture of all endmembers with equal abundances. By this way, the produced fractional abundances naturally satisfy the LMM with the nonnegative and sum-to-one constraints.

## Results

To test the performances of algorithms influenced by the noises, zero-mean Gaussian noises are added to the above two synthetic images to achieve different *signal-to-noise ratios* (SNRs) of 15, 25, 35, and 45 dB. The results with SREs obtained by the MCSUnADMM, CLSUnSAL, TVSUnSAL, and SUnSAL algorithms for these two synthetic images with different noise levels are reported in Table 1. As we can see, our proposed MCSUnADMM algorithm achieved the best unmixing accuracy than both SUnSAL and CLSUnSAL algorithms in two different simulated scenarios, and CLSUnSAL performs a little better than the SUnSAL algorithm. The TVSUnSAL algorithm has a little better performances for the SI-1 scene than that for the SI-2 scene. This may be because the two simulated scenes have different spatial characteristics (e.g., SI-1 are with a heterogeneous background while SI-2 has a relatively homogeneous background) while the TVSUnSAL algorithm aims at exploiting the spatial homogeneity to improve the unmixing accuracy. The improvement of MCSUnADMM demonstrates that the manifold regularization term is capable of enhancing the unmixing performance.

**Table 1:** SREs obtained by the MCSUnADMM, CLSUnSAl, TVSUnSAL, and SUnSAL algorithms on different SNRs

SNR (dB)	15	25	35	45
*SI-1*				
SUnSAL	2.302	4.262	8.851	16.035
CLSUnSAL	4.595	10.419	14.411	16.598
TVSUnSAL	2.104	4.212	8.564	15.091
MCSUnADMM	*6*.*519*	*13*.*924*	*19*.*763*	*21*.*144*
*SI-2*				
SUnSAL	1.516	2.415	5.184	13.032
CLSUnSAL	2.234	4.748	10.253	35.193
TVSUnSAL	2.670	*6*.*115*	15.926	38.323
MCSUnADMM	*2*.*673*	6.095	*16*.*023*	*38*.*517*

Best results are shown in italic

**Fig. 3:**
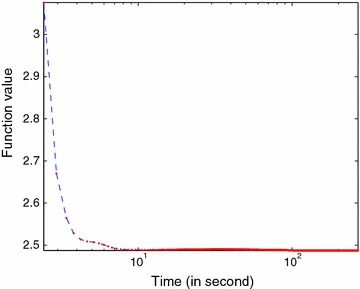
Evolution of the objective function (10) for SI-1 with $\hbox {SNR}=25\,\hbox {dB}$ by using MCSUnADMM algorithm

Table 2 reports the times of the MCSUnADMM, CLSUnSAL, TVSUnSAL, and SUnSAL algorithms for these two synthetic experiments with different SNRs when the stopping criterion (i.e., the maximum iteration number or the Frobenius norm of the pixel reconstruction errors, i.e. $\Vert {Y}-{A}{X}\Vert _F^2$, less than $\tau =1e^{-4}$) is reached. As can been see from this table, the proposed MCSUnADMM algorithm converges faster than the other three algorithms to reach the minima of the objective function $\Vert {Y}-{A}{X}\Vert _F^2$ because of the use of the locally geometrical structure of the hyperspectral data introduced by the manifold regularization. Note that a proper and efficient regularization introduced to the model will benefit the descending of the objective function. However, it must be pointed out that the computational complexity of solving model (10) is a little higher than solving the models (6) and (8) in theory. Table 3 gives the computational times for the SI-1 and SI-2 experiments by all of the four algorithms when setting the maximum iteration number (to be 2000) as the stopping criterion. According to this table, we can see that the SUnSAL and CLSUnSAL algorithms need almost the same computational times, while the TVSUnSAL and the MCSUnADMM algorithms have almost the same computational times, which are higher than that of the SUnSAL and CLSUnSAL algorithms. This is consistent with the theoretical analysis of the complexity (Iordache et al. 2012, 2014a). In addition, Fig. 3 plots the evolution of the objective function (10) versus time in the SI-1 experiments with $\hbox {SNR}=25\,\hbox {dB}$ to illustrate the convergence of the proposed MCSUnADMM algorithm.

**Table 2:** Times in second for the MCSUnADMM, CLSUnSAl, TVSUnSAL, and SUnSAL algorithms on different SNRs

Time (s)	15	25	35	45
*SI-1*				
SUnSAL	677	405	*194*	*133*
CLSUnSAL	575	225	213	203
TVSUnSAL	763	556	334	256
MCSUnADMM	*208*	*192*	195	194
*SI-2*				
SUnSAL	1136	1168	1091	1322
CLSUnSAL	1092	1136	1066	1916
TVSUnSAL	645	618	523	642
MCSUnADMM	*531*	*504*	*487*	*467*

Best results are shown in italic

**Table 3:** Times in second for the SI-1 and SI-2 experiments by the MCSUnADMM, CLSUnSAl, TVSUnSAL, and SUnSAL algorithms when setting the maximum iteration number as the stopping criterion

	SUnSAL	CLSUnSAL	TVSUnSAL	MCSUnADMM
SI-1	436	439	4242	4749
SI-2	754	763	20,496	20,512

**Fig. 4:**
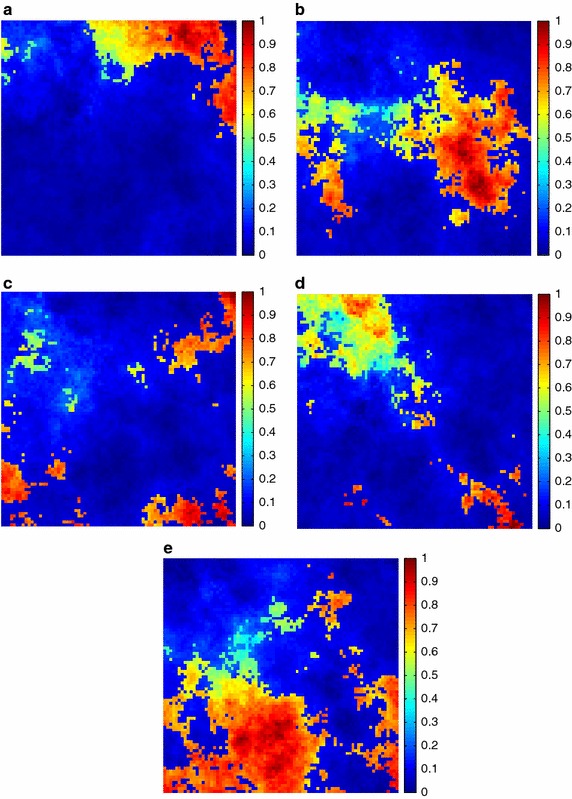
The ground-truth fractional abundance maps for SI-1. **a** Endmember 1, **b** Endmember 2, **c** Endmember 3, **d** Endmember 4, **e** Endmember 5

**Fig. 5:**
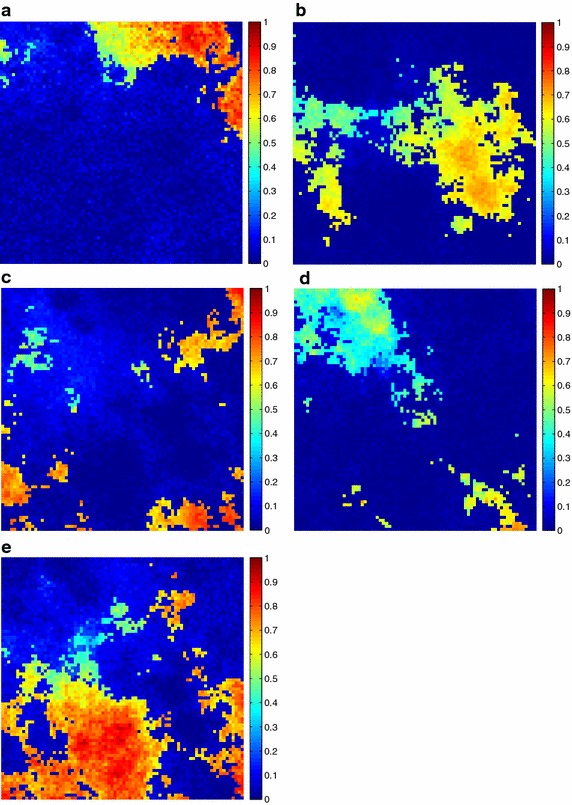
The recovered fractional abundance maps for SI-1 by the MCSUnADMM algorithm. **a** Endmember 1, **b** Endmember 2, **c** Endmember 3, **d** Endmember 4, **e** Endmember 5

**Fig. 6:**
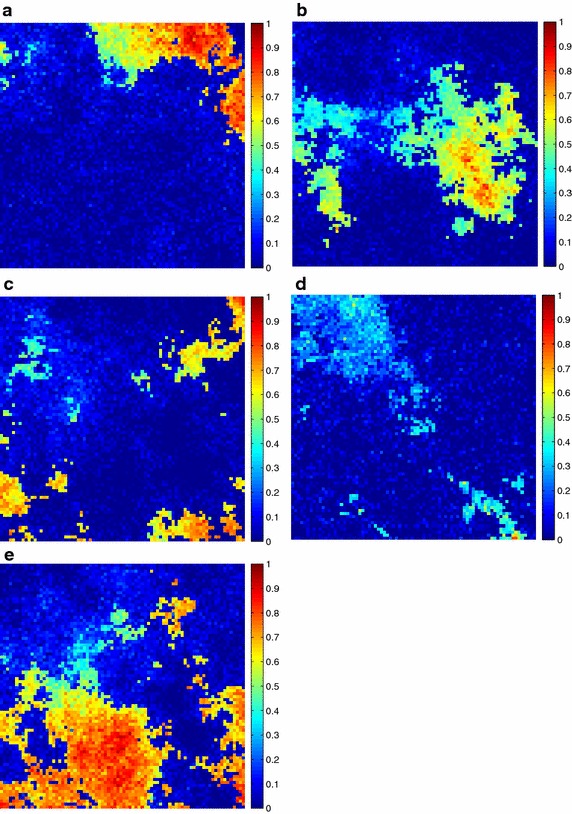
The recovered fractional abundance maps for SI-1 by the CLSUnSAL algorithm. **a** Endmember 1, **b** Endmember 2, **c** Endmember 3, **d** Endmember 4, **e** Endmember 5

**Fig. 7:**
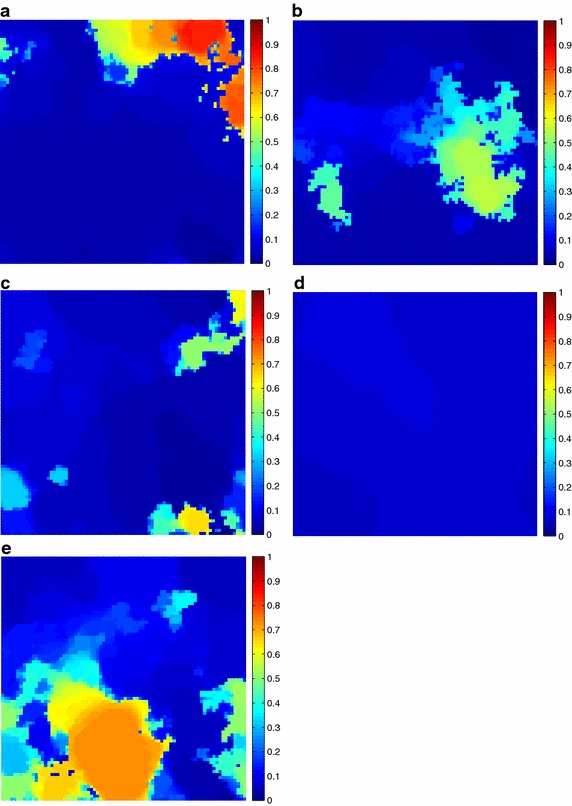
The recovered fractional abundance maps for SI-1 by the TVSUnSAL algorithm. **a** Endmember 1, **b** Endmember 2, **c** Endmember 3, **d** Endmember 4, **e** Endmember 5

**Fig. 8:**
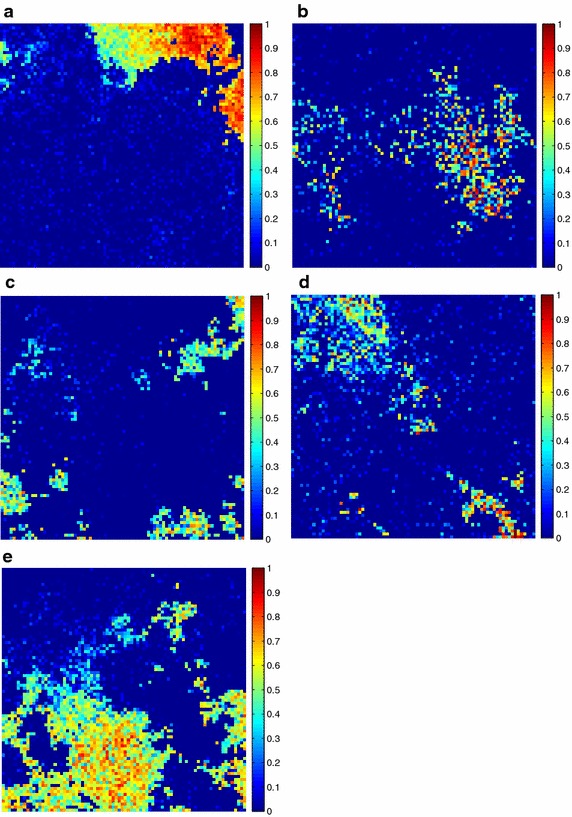
The recovered fractional abundance maps for SI-1 by the SUnSAL algorithm. **a** Endmember 1, **b** Endmember 2, **c** Endmember 3, **d** Endmember 4, **e** Endmember 5

**Fig. 9:**
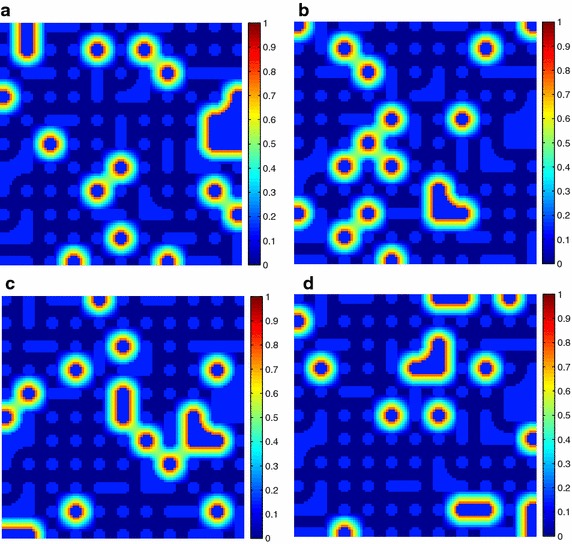
The ground-truth fractional abundance maps of endmember 1, 3, 5 and 7 for SI-2. **a** Endmember 1, **b** Endmember 3, **c** Endmember 5, **d** Endmember 7

**Fig. 10:**
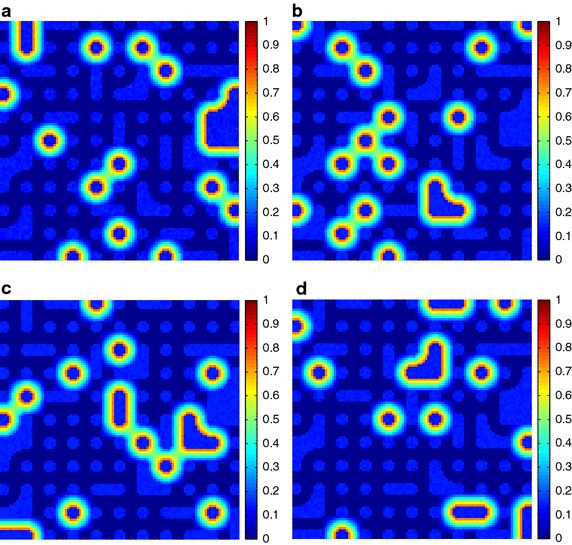
The recovered fractional abundance maps of endmember 1, 3, 5 and 7 for SI-2 by the MCSUnADMM algorithm. **a** Endmember 1, **b** Endmember 3, **c** Endmember 5, **d** Endmember 7

**Fig. 11:**
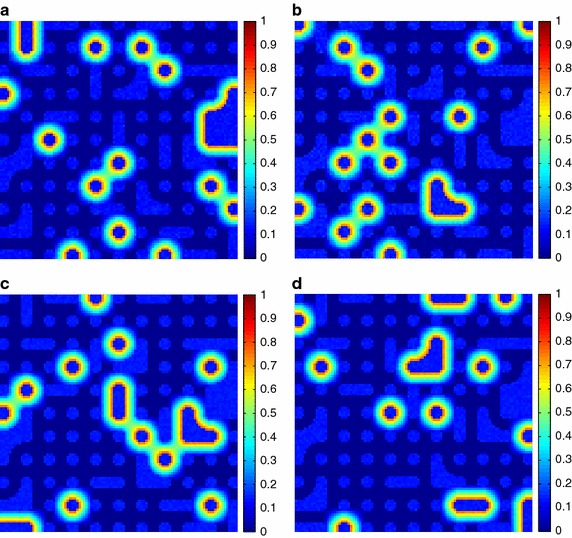
The recovered fractional abundance maps of endmember 1, 3, 5 and 7 for SI-2 by the CLSUnSAL algorithm. **a** Endmember 1, **b** Endmember 3, **c** Endmember 5, **d** Endmember 7

**Fig. 12:**
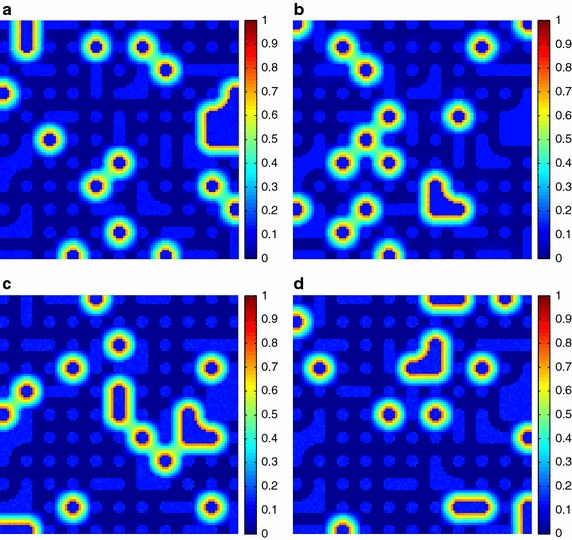
The recovered fractional abundance maps of endmember 1, 3, 5 and 7 for SI-2 by the TVSUnSAL algorithm. **a** Endmember 1, **b** Endmember 3, **c** Endmember 5, **d** Endmember 7

**Fig. 13:**
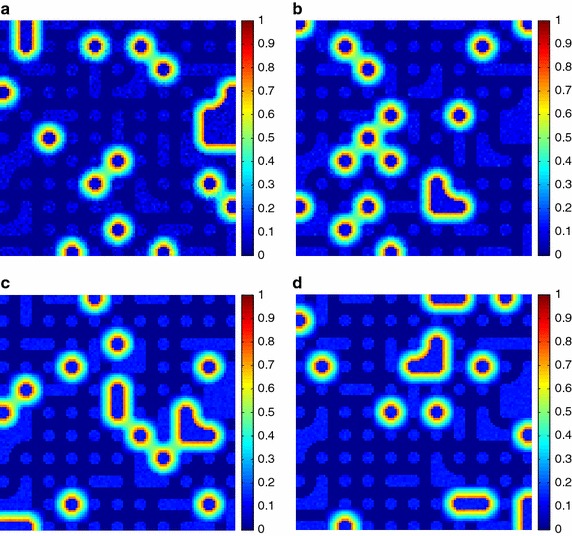
The recovered fractional abundance maps of endmember 1, 3, 5 and 7 for SI-2 by the SUnSAL algorithm. **a** Endmember 1, **b** Endmember 3, **c** Endmember 5, **d** Endmember 7

Additionally, the estimated fractional abundances obtained by the three algorithms, along with the ground-truth abundances, are shown in Figs. 4, 5, 6, 7, 8, 9, 10, 11, 12 and 13. For space consideration, only the fractional abundance maps of SI-1 with the lowest SNR of 15 dB and the fractional abundance maps of SI-2 with the highest SNR of 45 dB are reported. By visual comparisons of these fractional abundance maps, it can be seen that our proposed MCSUnADMM algorithm based on MCS model outperforms the other two algorithms for SR and CSR models and the incorporated manifold regularization can impose spatial consistency such that the spatially similar pixels have similar abundances as shown in Figs. 5, 6, 7, 8, 9 and 10. From Fig. 7, we can see that the results obtained by the TVSUnSAL algorithm exhibits more spatial homogeneity due to the total variation regularization. However, the proposed MCSUnADMM algorithm can deal with the pixels in the transaction areas, as shown in Fig. 5, since its incorporated manifold regularization has applied different weights (9) for different pixels.

## Real hyperspectral image experiments

In this section, we apply the proposed MCSUnADMM algorithm to real hyperspectral data collected by the Airborne Visible/InfRared Imaging Spectrometer (AVIRIS). The AVIRIS instrument can cover a spectral region from 0.41 to 2.45 μm in 224 bands with a 10 nm bandwidth.

### Data sets

**Fig. 14:**
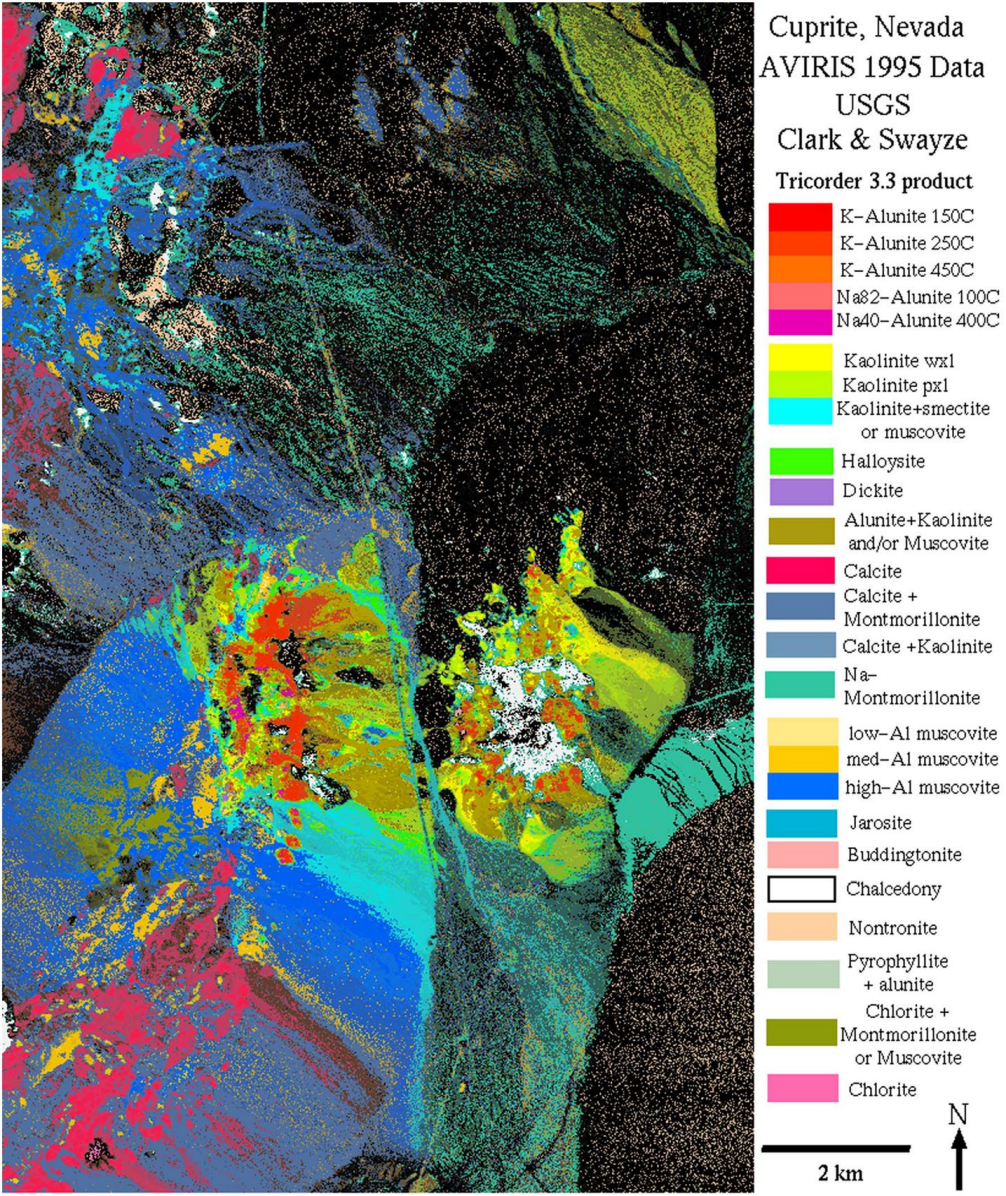
The mineral abundance map of the Cuprite mining site produced in 1995 by USGS

The studied real hyperspectral image is collected by AVIRIS over the Cuprite mining site, Nevada, on June 19, 1997 and is publicly available online. The Cuprite data contains some exposed minerals included in the above used USGS spectral library ${A}$, and is well understood mineralogically. The mineral map produced by USGS is shown in Fig. 14. It should be noted that we only exhibit this figure as a reference to visually compare the results obtained by different methods, since the mineral map was produced in 1995 while the Cuprite data was collected in 1997 (Iordache et al. 2014a). This scene is often used for the assessment of the abundance maps obtained by spectral unmixing algorithms (Iordache et al. 2012).

### Results analysis

We conduct our experiment on a subscene with a size of $250\times 190$ pixels and 224 bands. However, the bands 1–2, 104–113, 148–167, and 221–224 were removed due to water absorption and noise, and thus only a total of 188 bands were used in the experiment. In addition, the corresponding water absorption and noise bands are also removed from the spectral library ${A}$.

**Fig. 15:**
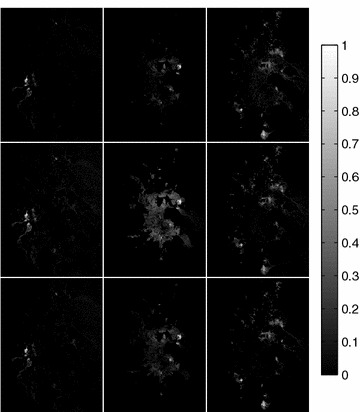
The estimated fractional abundance maps by the MCSUnADMM (*first row*), CLSUnSAL (*second row*), and SUnSAL (*third row*) methods for the subscene of AVIRIS Cuprite data

The fractional abundance maps of three typical minerals estimated by the MCSUnADMM, CLSUnSAL, and SUnSAL methods are shown in Fig. 15. By visually comparison, we can find that the abundance maps obtained by our proposed MCSUnADMM method are more sparse and have less outliers than that of the CLSUnSAL and SUnSAL methods, this may be due to the incorporated manifold regularization can consider the geometric structure of the dataset such that the performances of the MCSUnADMM method exhibit good spatial consistency.

## Conclusions

This paper presents a novel sparse unmixing model, which incorporate the manifold regularization into the collaborative sparse regression. The manifold regularization is induced by a Laplacian graph, which can characterize the locally geometrical structure of the hyperspectral data. In this way, the proposed manifold regularized collaborative sparse (MCS) model can consider both the Euclidean structures and underlying manifold structures. To solve the proposed model, an efficient algorithm based on ADMM, called MCSUnADMM, has been developed. And the convergence of the proposed MCSUnADMM algorithm can be guaranteed based on the framework of ADMM. Experimental results with both simulated and real hyperspectral datasets demonstrate the effectiveness of the proposed model and algorithm. However, the efficiency of the proposed MCSUnADMM algorithm is affected by two regularization parameters. Therefore, our future work will focus on designing a strategy to adaptively set these parameters. In addition, further experiments with different scenes of real hyperspectral images are need to investigate the performances of our proposed model and method.

## References

[CR1] Afonso MV, Bioucas-Dias JM, Figueiredo MAT (2011). An augmented Lagrangian approach to the constrained optimization formulation of image inverse problems. IEEE Trans Image Process.

[CR3] Belkin M, Niyogi P (2003). Laplacian eigenmaps for dimensionality reduction and data representation. Neural Comput.

[CR2] Belkin M, Niyogi P, Sindhwani V (2006). Manifold regularization: a geometric framework for learning from labeled and unlabeled examples. J Mach Learn Res.

[CR4] Bioucas-Dias J, Figueiredo M (2010) Alternation direction algorithms for constrained sparse regression: application to hyperspectral unmixing. In: Proceedings of 2nd workshop hyperspectral image signal processing—evolution in remote sensing

[CR5] Bioucas-Dias JM, Plaza A (2012). Hyperspectral unmixing overview: geometrical, statistical, and sparse regression-based approaches. IEEE J Sel Top Appl Earth Obs Remote Sens.

[CR6] Bioucas-Dias JM, Plaza A (2013). Hyperspectral remote sensing data analysis and future challenges. IEEE Geosci Remote Sens Mag.

[CR7] Boardman JM, Kruse FA, Green RO (1995) Mapping target signatures via partial unmixing of aviris data. In: Proceedings of summer JPL ariborne earth science workshop, Pasadena, CA

[CR8] Boris K (1999) Computations with Gaussian random fields. PhD thesis, Institute for System Research, University of Maryland

[CR9] Cai D, He X, Han J, Huang TS (2011). Graph regularized nonnegative matrix factorization for data representation. IEEE Trans Pattern Anal Mach Intell.

[CR10] Chan TH, Ma WK, Ambikapathi A, Chi CY (2011). A simplex volume maximization framework for hyperspectral endmember extraction. IEEE Trans Geosci Remote Sens.

[CR13] Chang CI, Heinz DC (2000). Constrained subpixel target detection for remotely sensed imagery. IEEE Trans Geosci Remote Sens.

[CR11] Chang CJ (2006). A new growing method for simplex-based endmember extraction algorithm. IEEE Trans Geosci Remote Sens.

[CR12] Chang CI, Wu CC, Chen HM (2010). Random pixel purity index. IEEE Geosci Remote Sens Lett.

[CR14] Charles AS, Olshausen BA, Rozell CJ (2011). Learning sparse codes for hyperspectral imager. IEEE J Sel Top Signal Process.

[CR15] Chen X (2011). A quantitative analysis of virtual endmembers in creased impact on the collinearity effect in spectral unmixing. IEEE Trans Geosci Remote Sens.

[CR16] Donoho D, Grimes C (2003). Hessian eigenmaps: locally linear embedding techniques for high dimensional data. Proc Natl Acad Sci.

[CR17] Eckstein J, Bertsekas D (1992). On the Douglas–Rachford splitting method and the proximal point algorithm for maximal monogone operators. Math Program.

[CR18] Fan W, Hu B, Miller J, Li M (2009). Comparative study between a new nonlinear model and common linear model for analysing laboratory simulated-forest hyperspectral data. Int J Remote Sens.

[CR19] Feng R, Zhong Y, Zhang L (2014). Apdative non-local euclidean medians sparse unmixing for hyperspectral imagery. ISPRS J Photogramm Remote Sens.

[CR20] Guan N, Tao D, Luo Z, Yuan B (2011). Manifold regularized discriminative nonnegative matrix factorization with fast gradient descent. IEEE Trans Image Process.

[CR21] Hapke B (1981). Bidirectional reflectance spectroscopy 1. Theory. J Geophys Res.

[CR24] He X, Niyogi P (2004). Locality preserving projections. Adv Neural Inf Process Syst.

[CR22] He X, Cai D, Shao Y, Bao H, Han J (2011). Laplacian regularized gaussian mixture model for data clustering. IEEE Trans Knowl Data Eng.

[CR23] Heinz D, Chang CI (2001). Fully constrained least squares linear mixture analysis for material quantification in hyperspectral imagery. IEEE Trans Geosci Remote Sens.

[CR25] Iordache MD, Bioucas-Dias JM, Plaza A (2011). Sparse unmixing of hyperspectral data. IEEE Trans Geosci Remote Sens.

[CR26] Iordache MD, Bioucas-Dias JM, Plaza A (2012). Total variation spatial regularization for sparse hyperspectral unmixing. IEEE Trans Geosci Remote Sens.

[CR27] Iordache MD, Bioucas-Dias JM, Plaza A (2014). Collaborative sparse regression for hyperspectral unmixing. IEEE Trans Geosci Remote Sens.

[CR28] Iordache MD, Bioucas-Dias JM, Plaza A (2014). Music-CSR: hyperspectral unmixing via multiple signal classification and collaborative sparse regression. IEEE Trans Geosci Remote Sens.

[CR29] Keshava N (2003). A survey of spectral unmixing algorithms. Linc Lab J.

[CR30] Lin T, Zha H (2008). Riemannian manifold learning. IEEE Trans Pattern Anal Mach Intell.

[CR31] Liu JM, Zhang JS (2012). A new maximum simplex volume method based on householder transformation for endmember extraction. IEEE Trans Geosci Remote Sens.

[CR32] Liu J, Zhang J (2014). Spectral unmixing via compressive sensing. IEEE Trans Geosci Remote Sens.

[CR33] Lu X, Wu H, Yuan Y, Yan P, Li X (2013). Manifold regularized sparse nmf for hyperspectral unmixing. IEEE Trans Geosci Remote Sens.

[CR34] Ma W, Bioucas-Dias JM, Chan TH (2014). A signal processing perspective on hyperspectral unmixing: insights from remote sensing. IEEE Signal Process Mag.

[CR35] Meer FVD (2012). Remote-sensing image analysis and geostatistics. Int J Remote Sens.

[CR36] Miao L, Qi H (2007). Endmember extraction from highly mixed data using minimum volume constrained nonnegative matrix factorization. IEEE Trans Geosci Remote Sens.

[CR37] Nascimento J, Bioucas-Dias JM (2005). Vertex component analysis: a fast algorithm to unmix hyperspectral data. IEEE Trans Geosci Remote Sens.

[CR38] Nascimento JMP, Bioucas-Dias M (2012). Hyperspectral unmixing based on mixtures of Dirichlet components. IEEE Trans Geosci Remote Sens.

[CR39] Pu H, Xia W, Wang B, Jiang G (2014). A fully constrained linear spectral unmixing algorithm based on distance geometry. IEEE Trans Geosci Remote Sens.

[CR40] Roweis S, Saul L (2000). Nonlinear dimensionality reduction by locally linear embedding. Science.

[CR41] Settle JJ, Drake NA (1993). Linear mixing and the estimation of ground cover proportions. Int J Remote Sens.

[CR42] Seung HS, Lee DD (2000). The manifold ways of perception. Science.

[CR43] Shaw GA, Burke HK (2003). Spectral imaging for remote sensing. Linc Lab J.

[CR44] Shimabukuro YE, Carvalho VC, Rudorff BFT (1997). NOAA-AVHRR data processing for the mapping of vegetation cover. Int J Remote Sens.

[CR45] Singer RB, Mccord TB (1979) Mars: large scale mixing of bright and dark surface materials and implications for analysis of spectral reflectance. In: Proceedings of 10th lunar planetary science conference, Washington, DC

[CR47] Somers B, Delalieux S (2009). A weighted linear spectral mixture analysis approach to address endmember variability in agricultural production systems. Int J Remote Sens.

[CR46] Somers B, Asnerc GP, Tits L, Coppin P (2011). Endmember variability in spectral mixture analysis: a review. Remote Sens Environ.

[CR48] Stein D (2003) Application of the normal compositional model to the analysis of hyperspectral imagery. In: Proceedings of IEEE workshop advances in techniques for analysis of remotely sensed data (2003)

[CR49] Tang W, Shi Z, Wu Y, Zhang C (2015). Sparse unmixing of hyperspectral data using spectral a priori information. IEEE Trans Geosci Remote Sens.

[CR50] Tenenbaum JB, Silva V, Landford JC (2000). A global geometric framework for nonlinear dimensionality reduction. Science.

[CR51] Tong L, Zhou J, Bai X, Gao Y (2014) Dual graph regularized NMF for hyperspectral unmixing. In: Digital image computing techniques and applications

[CR52] Winter ME (1999) N-findr: an algorithm for fast autonomous spectral endmember determination in hyperspectral data. In: Proceedings of SPIE conference on imaging spectrometry, Pasadena, CA

[CR53] Yang JF, Zhang Y (2011). Alternating direction algorithms for $$\ell _1$$-problems in compressive. SIAM J Sci Comput.

[CR55] Zare A, Ho KC (2014). Endmember variability in hyperspectral analysis: addressing spectral variability during spectral unmixing. IEEE Signal Process Mag.

[CR54] Zare A, Gader P, Dranishnikov D, Glenn T (2013) Spectral unmixing using the beta compositional model. In: Proceedings of IEEE workshop. Hyperspectral image and signal processing: evolution in remote sensing, Gainesville, FL

[CR56] Zheng M, Bu J, Chen C, Wang C, Zhang L, Qiu G, Cai D (2011). Graph regularized sparse coding for image representation. IEEE Trans Image Process.

[CR57] Zhong Y, Zhang L (2014). Non-local sparse unmixing for hyperspectral remote sensing imagery. IEEE J Sel Top Appl Earth Obs Remote Sens.

